# Implementation of the infection control estimate: A case study on the use of a newly developed digital tool for outbreak management in the acute setting

**DOI:** 10.1177/17571774221127576

**Published:** 2022-09-15

**Authors:** Matthew Oliver Wynn, Sandra Brady, Josh McKenna, Linda Swanson, Ryan George

**Affiliations:** 1 School of Health and Society, 7046 University of Salford, UK; 2 Infection Control, 523611 Northern Care Alliance NHS Foundation Trust, UK; 3 Infection Control/Tissue Viability, 5293 Manchester University NHS Foundation Trust, UK

**Keywords:** Outbreaks, ergonomics, infection control estimate, planning, digital

## Abstract

**Aim:**

An Infection Control Estimate (ICE) Tool was developed based on a previously published concept of applying military planning techniques to Infection Prevention and Control (IPC) management strategies in the acute healthcare setting.

**Methods:**

Initial testing of the outbreak management tool was undertaken in a large acute hospital in the North-West of England during a localised outbreak of COVID-19. The tool, developed using Microsoft Excel, was completed by trained IPC practitioners in real-time to log outbreak details, assign and manage meeting actions and to generate surveillance data.

**Results:**

The ICE tool was utilised across five outbreak control meetings to identify and allocate tasks to members of the outbreak control team and to monitor progress. Within the meetings, the tool was used primarily by the trained IPC Specialist Nurses who were guided by and entered data into the relevant sections. Feedback indicated that the tool was easy to use and useful as the sole repository of outbreak information and data. Suggested improvements following the testing period were made and additional functionality was added.

**Conclusion:**

Utilisation of the ICE tool has the potential to improve our understanding of the efficacy of currently employed outbreak management interventions and provides a cognitive support and targeted education for teams responsible for the management of outbreaks. It is hoped that by guiding teams through an outbreak with prompts and guidance, as well as facilitating collection and presentation of surveillance data, outbreaks will be resolved sooner and risks to patients will be reduced.

## Introduction

The COVID-19 pandemic generated numerous challenges to healthcare services and has in many cases hastened a transition towards digital ways of working. These challenges highlighted several key issues related to the management of outbreaks of infectious diseases. Firstly, an appreciation of the importance of robust and timely datasets to support decision making has been realised during contact tracing and epidemiological modelling efforts (Budd et al., 2020). Secondly, the paramount importance of human factors and ergonomics, ensuring that workflows and processes are effective, engaging and barriers to outbreak interventions are recognised and addressed (Gurses et al., 2020).

The most recent Centre for Workforce Intelligence review of the IPC (Infection Prevention and Control) workforce highlighted those approaches to infection control service delivery, including outbreak management, are varied and there is little consistency in the training, practice or philosophies underlying IPC services in the UK (United Kingdom) (CFWI 2015). In response to challenges associated with the management of outbreaks in acute inpatient settings, particularly of the scale and complexity seen during the early months of the COVID-19 pandemic, a new process was promptly developed. The Infection Control Estimate (ICE) is an adapted version of a process used by the British armed forces, the ‘Combat Estimate’ (Wynn et al., 2020). The process requires consideration of seven broad questions which help focus clearly on the issue at hand and how it can be solved in a timely and effective manner. See Table 1 which illustrates the adaptation of the CE into the ICE.

**Table 1.** Generation of the infection control estimate.

Combat estimate	Infection control estimate	Purpose	Traditional elements of outbreak management
			Identification
What are the enemy doing and why?	What is the nature, distribution, and mode of transmission of the organism?	Situation analysis	Investigation
What have I been told to do and why?	What is the objective/why do I need to control this organism?		Case definition
What actions/effects do I want to have on the enemy?	How can I reduce the risk of onward transmission/limit impact to patients?		Control measures
Where can I best accomplish each action/effect?	Where and when will each of my control actions be most effective?	Planning	
What resources do I need to accomplish each action/effect?	What resources will I require to achieve my aims and who will be most effective in delivering these?		
When and where do these actions take place in relation to each other?	In what order will I perform my control plan and where will be best to focus efforts for maximum impact?		
What control measures do I need to impose?	What are the interventions/what post-action evaluation do I need to impose to determine if my plan was effective?	Evaluation	Communication

This article will discuss the first proactive application of this novel outbreak management tool during an outbreak of COVID-19 in a large acute hospital in the North-West of England.

In a military context, the CE questions are typically used alongside an *aide memoir* as a cognitive support to ensure that key elements of planning are not missed. In the context of the ICE, a prototype digital tool was developed using Microsoft Excel and contains data entry fields and guiding questions. The tool supports the development, monitoring, and allows retrospective analysis of the efficacy of outbreak management efforts by facilitating data extraction with potential to be used as metric for outbreak management efficacy and quality. Table 2 illustrates the data captured by the ICE tool.

**Table 2.** Key data captured by the ICE tool.

Data	Potential value
Case definitions	• Supports all clinical staff with access to the tool to accurately identify affected patients and reduce the risk of missed cases
Outbreak management objectives	• Supports objective-oriented decision making
Outbreak management team structure	• Supports staff to identify relevant individuals to raise concerns/issues with
Outbreak timeline (with annotations including interventions)	• Retrospective analysis of intervention efficacy
	• Real-time visual indications of intervention efficacy
Relevant transmission data related to the causative organism	• Supports non-experts to identify potential interventions to break the chain of infection during outbreak management meetings
	• Eliminates the risk of OMT members making assumptions about transmission dynamics
Attendance at outbreak management meetings, plus contact details	• Allows ownership/oversight and rapid/automated distribution of communications related to the outbreak
Interventions implemented with times and dates of implementation	• May contribute to understanding of the efficacy of outbreak management interventions and support the development of future artificial intelligence (AI) technologies to support planning during outbreak management efforts
	• Provides cognitive support to prompt follow up and evaluation of outbreak interventions
	• Acts as an audit log of actions taken
Advice given by IPC team	• Secondary analysis of information needs in relation to IPC among frontline clinical staff/direct future education requirements
Resources required for outbreak interventions	• May support economic analyses on the impacts of outbreaks/requirements for IPC team staffing and budget
Interventions utilised stratified by position in the hierarchy of controls	• Allows evaluation of the potential impacts/appropriateness of outbreak interventions to be undertaken
Negative impacts of interventions implemented (e.g. nursing male and female patients in the same bay due to the closure of clinical areas)	• Allows evaluation of the negative impacts of outbreak interventions to be undertaken more clearly to support decision making

### Background of outbreak

In 2021, the ICE protype tool was used for the first time during a live outbreak at a hospital in Northwest England. The outbreak concerned SARS-COV-2 and lasted 44 days, during which time 36 patients were infected. The outbreak was contained to a single ward.

### The ICE prototype

**Figure 1.**
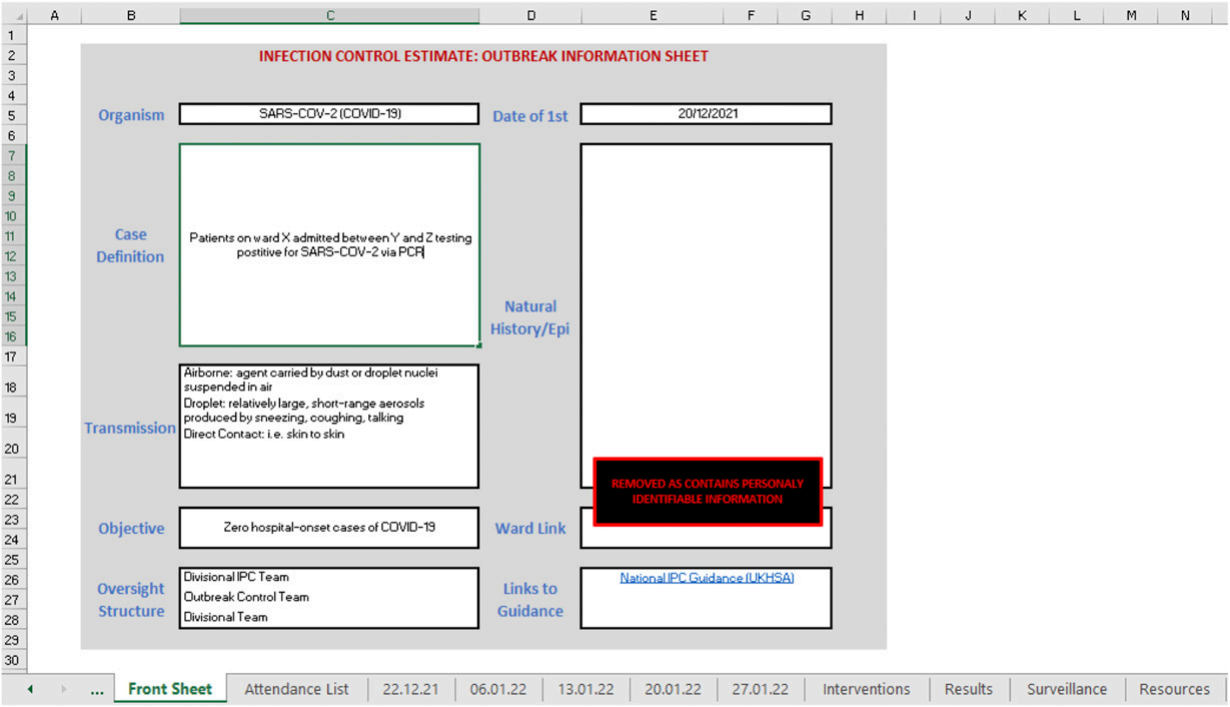
The ICE prototype front sheet/user interface.

**Figure 2.**
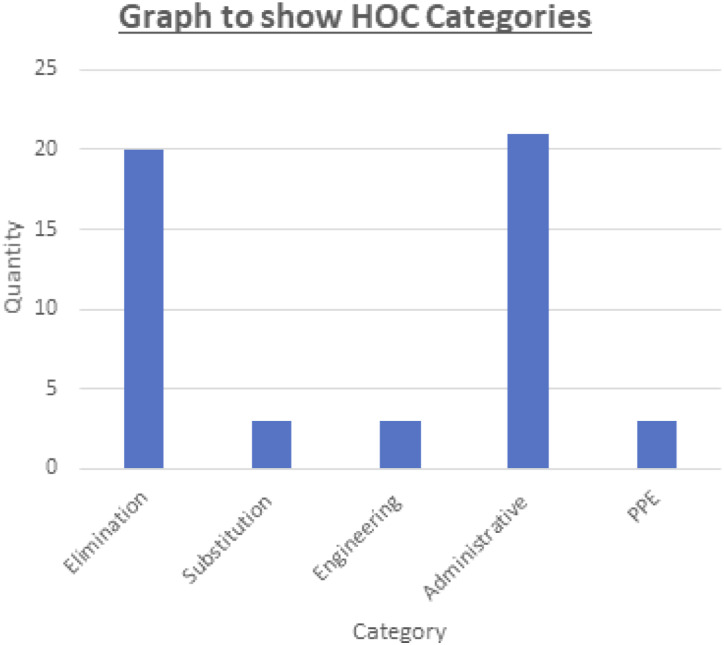
Hierarchy of control distribution for interventions used during the outbreak.

**Figure 3.**
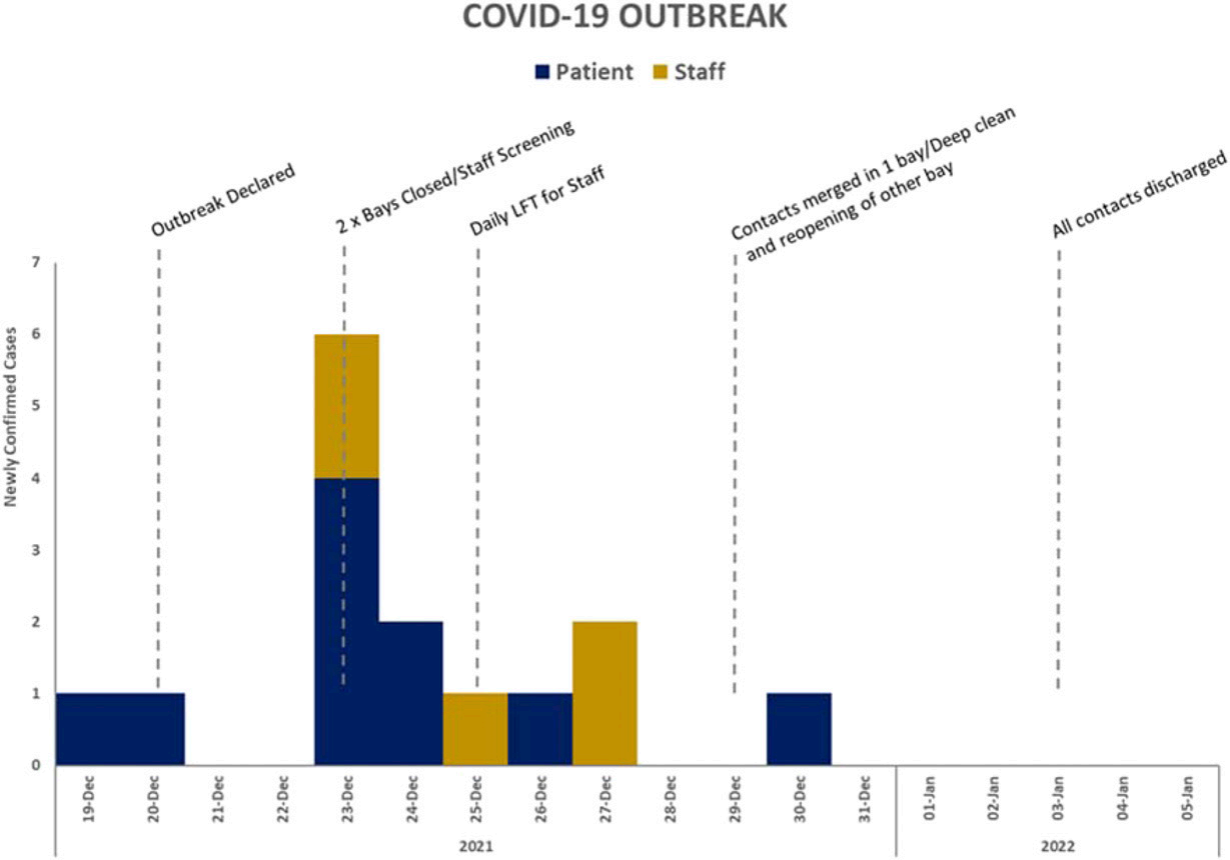
Visualisation of the outbreak using epidemiological and intervention implementation data.

The ICE tool prototype was used to document information related to the outbreak and answers the seven questions which constitute the estimate (Wynn et al., 2020). For example, the front sheet, as seen in Figure 1, contains data related to the causative organism, its modes of transmission, a case definition and an epidemic curve which addresses question one (What is the nature, distribution and mode of transmission of the organism?). In addition to providing a clear objective for outbreak management addressing question two (What is the objective/why do I need to control this organism?). Following the initial development of the prototype tool, it was tested using data from a historical outbreak to help identify gaps in the data collection or additional data entry fields which may support the management of outbreaks which could be included. Figures 2 and 3 show data visualisation capabilities of the ICE prototype.

Throughout the outbreak, the ICE prototype tool was used to record key data related to the outbreak in addition to being used to help plan and evaluate control measures. A retrospective analysis of the ICE tool used during the outbreak identified 53 interventions employed. These were automatically analysed by the tool and categorised into the relevant hierarchy of control (HOC) categories (Centers for Disease Control 2015). This analysis highlighted that the majority (*n* = 41) of outbreak interventions were either elimination or administrative interventions with the minority of interventions (*n* = 12) representing substitution, engineering controls or personal protective equipment-based interventions.

The tool was utilised across five outbreak control meetings to identify and allocate tasks to members of the outbreak control team. The tool also indicates when these tasks were completed. Within the meetings the ICE tool was used primarily by the trained infection control specialist nurses who were guided by and entered data into the tool. It was reported that the tool was found to be easy to use and captured all necessary data, although new data collection fields were suggested during the outbreak including detailed recording of issues and negative impacts of management interventions.

## Conclusion

The ICE prototype is the first digital tool which centralises key data related to the management of outbreaks in the context of inpatient hospital settings. The data captured within the ICE tool has the potential to improve our understanding of the efficacy of currently employed outbreak management interventions, the economic impacts of outbreaks and provides a cognitive support and targeted educational tool for teams responsible for the management of outbreaks. By ensuring that all aspects of the ICE seven questions are addressed within the tool, teams can be more confident that all key elements of the outbreak control process have been considered and all potential interventions have been implemented and evaluated. Future development of the tool will include the addition of greater communication functionality in addition to automated analysis of data captured within the tool to help better understand the efficacy of interventions and facilitate rapid and effective dissemination, and archiving, of the outbreak management plans to relevant clinical staff and leaders. Whilst this initial pilot testing of the tool during an outbreak has demonstrated the basic usability of the tool, wider robust testing is required to evaluate the value and potential impact of the ICE on outbreak management processes.
